# Artificial Intelligence in Hematologic Malignancies: Opportunities, Challenges, and Clinical Integration

**DOI:** 10.7759/cureus.100950

**Published:** 2026-01-06

**Authors:** Aya Salem, Mahmoud Teama, Hassan A Kassem, Niyousha Vakilzadehian, Arig Mahmoud Abdelaziz Ali, Dharaneswar Venugopal, Ahmed M. Khalifa

**Affiliations:** 1 Dermatology and Venerology, El Anfoushi Children Hospital, Alexandria, EGY; 2 General Medicine, King’s College Hospital NHS Foundation Trust, London, GBR; 3 Critical Care, King’s College London, London, GBR; 4 General Internal Medicine, King’s College London, London, GBR; 5 Gastroenterology and Hepatology, Cairo Fatemic Hospital, Cairo, EGY; 6 Neuro Rehabilitation, King’s College Hospital NHS Foundation Trust, London, GBR; 7 Cardiology, Frimley Park Hospital, Frimley Health NHS Foundation Trust, Frimley, GBR

**Keywords:** artificial intelligence, deep learning, hematologic malignancies, leukemia, lymphoma, machine learning, multiple myeloma, personalized medicine, prognosis, radiomics

## Abstract

Artificial intelligence (AI) has shown significant potential in enhancing diagnostic accuracy, refining prognostic models, guiding personalized treatment decisions, and improving clinical workflows. Across the spectrum of blood cancers, AI-based tools have demonstrated strong performance in tasks such as automated image classification, genomic and biomarker analysis, prediction of treatment response and toxicity, and estimation of measurable residual disease. Despite these promising developments, challenges remain, including limited dataset size, lack of prospective validation, concerns regarding interpretability, and ethical considerations related to data privacy and bias. The review emphasizes the need for robust clinical integration strategies, high-quality data, and multiple teams' collaboration to fully harness AI’s potential in transforming the management of hematologic malignancies.

## Introduction and background

Hematologic malignancies comprise a diverse group of cancers that originate from cells within the blood-forming system, including leukemia, lymphoma, and myeloma [1]. These conditions present clinically with signs of insufficient or suppressed bone marrow function, including a propensity for bleeding, susceptibility to infections, and anemia [2,3].

The rapid advancement of computer science over the last decade has led to the widespread integration of artificial intelligence (AI) as a standard feature across various sectors, most notably within healthcare [4]. AI is a computer program that enables machines to simulate certain human thought processes and intelligent behaviors, helping to solve complex problems. Machine learning (ML), a subset of AI, is a valuable supportive tool that seeks out comparable patterns within datasets. These capabilities enable the creation of various models for accomplishing complex tasks. Exciting areas of development in AI include natural language processing (NLP), large language models (LLMs), explainable AI (XAI), and generative AI. While these are all interconnected, each branch or technology possesses distinct characteristics and fields of application [5].

ML tools show great promise across many medical disciplines [6-9], particularly in oncology. Various algorithms have been suggested for applications like assessing cancer risk, automating segmentation, identifying and characterizing lesions, determining grading and staging, and predicting patient outcomes and treatment effectiveness [10]. This study aims to evaluate the role of artificial intelligence in hematologic malignancies by summarizing current applications of machine learning across leukemia, lymphoma, and multiple myeloma for diagnosis, prognosis, and treatment optimization, while highlighting clinical benefits, limitations, and future requirements for safe integration of AI-driven tools into routine hematology practice and improving personalized, data-driven patient care.

## Review

The need for AI in hematologic malignancies

AI has been an area of research for some time, but recent advances, largely due to better access to data and enhanced computing capabilities, suggest this fast-developing field could potentially revolutionize society [11,12].

AI has advanced significantly in many fields, including medicine, over the last decade. Hematology and oncology are data-intensive areas undergoing rapid innovation, with a strong clinical need for improved efficiency and advanced tools for diagnosis and treatment planning. The rising global elderly population suggests an increase in cancer incidence. Simultaneously, our abilities to diagnose and treat cancer are constantly improving. These trends lead to a massive increase in data and more complicated clinical processes, a complexity further amplified by advancements in all related medical specialties, such as hematology, oncology, radiology, pathology, surgery, human genetics, and nuclear medicine. The variability among patients demands customized approaches, necessitating the creation of new scientific methods [13].

**Figure 1:**
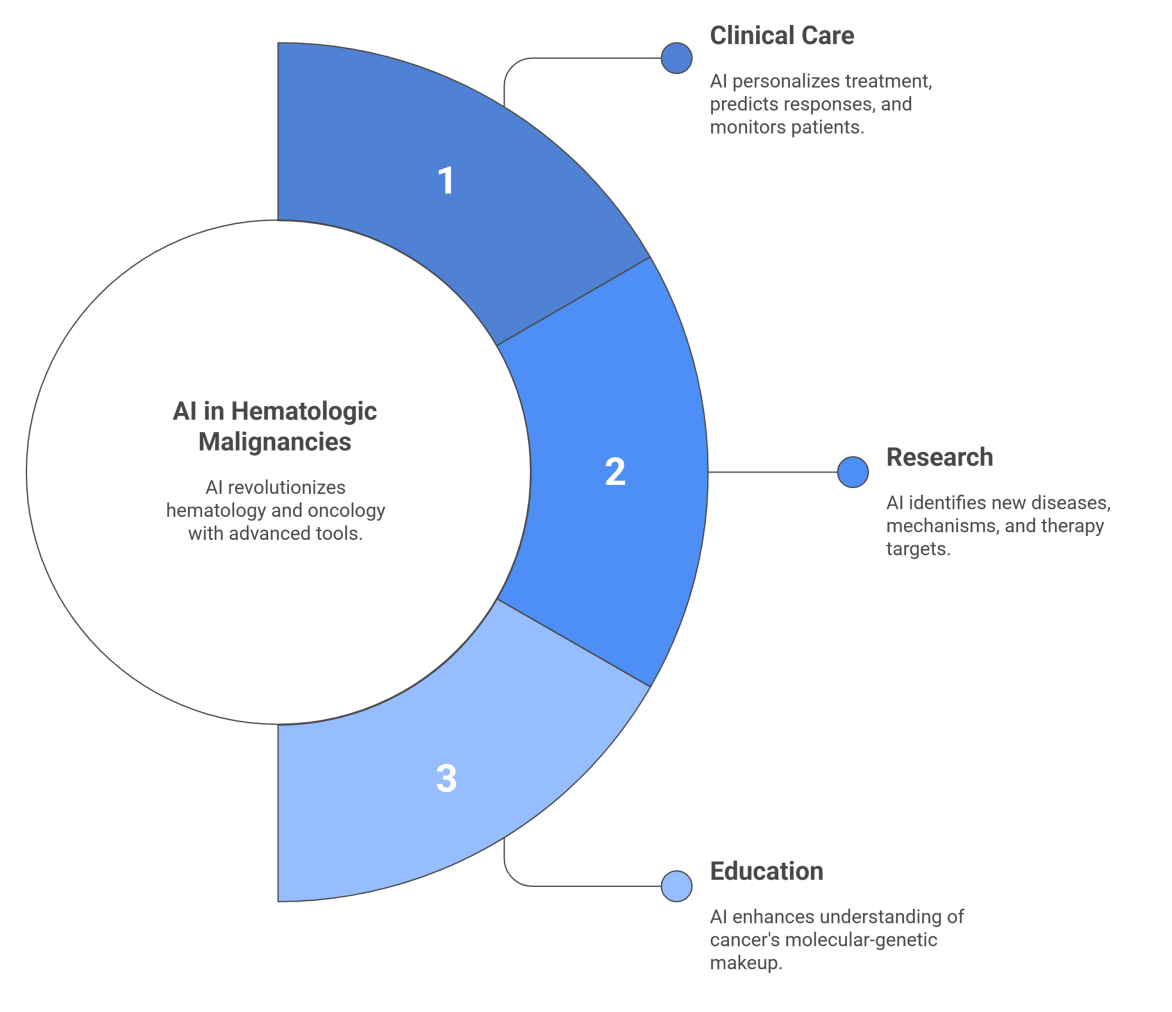
The need for AI in hematologic malignancies. Image credit: Created by the article authors based on [10-16].

AI has three main applications: clinical care (personalized), research, and education. We focus primarily on the clinical application because it directly benefits patients and doctors in hematology and oncology. For instance, AI can analyze past cases to predict a patient's response to a specific treatment, offer treatment suggestions based on individual tumor traits, and monitor patients. Beyond clinical use, AI serves as a powerful research tool, helping to derive new scientific knowledge from clinical data, such as identifying new disease types or underlying mechanisms. In the future, this could lead to a better understanding of cancer's molecular-genetic and cellular makeup, new uses for existing drugs, uncovering hidden patterns in image data, identifying novel therapy targets or pathological processes, or discovering new biomarkers (Figure 1) [13-16].

Leukemia

Leukemia is a blood cancer marked by the excessive and unchecked growth of immature white blood cells, known as blasts. It is categorized as either acute or chronic depending on how quickly the disease advances. Acute leukemia is a rapidly progressing and aggressive disease where a swift increase in blast cells can be fatal if treatment is delayed. Conversely, chronic leukemia typically develops more gradually [17-20]. Leukemia is categorized into four main types based on whether the affected cells are myeloid or lymphoid and whether the disease is acute or chronic: acute lymphoblastic leukemia (ALL), chronic lymphoid leukemia (CLL), acute myeloid leukemia (AML), and chronic myeloid leukemia (CML) [18,21,22].

Diagnosing leukemia is an inherently complex and often time-consuming procedure. Consequently, the implementation of ML techniques presents a significant opportunity to provide valuable assistance and potentially streamline this critical diagnostic phase [23]. ML presents a hopeful method for managing leukemia because it has the potential to detect genetic changes specific to the disease [24]. The most commonly employed ML techniques in this area include the least absolute shrinkage and selection operator (LASSO), random forest (RF) graph, support vector machine (SVM), and decision tree algorithms [25]. Numerous scientific articles report the application of ML algorithms to enhance diagnostic accuracy, predict outcomes, and guide treatment plans for patients suffering from various forms of leukemia [26].

Early research applying ML in leukemia primarily involved identifying leukemic cells in blood samples, analyzing flow cytometry data, and assessing genetic information. ML is capable of precisely categorizing leukemia subtypes. For example, Ahmed et al. employed a convolutional neural network (CNN), a form of deep learning (DL), to automatically distinguish between AML, CML, ALL, and CLL. This CNN algorithm demonstrated high accuracy, achieving 88.25% for leukemia diagnosis and 81.74% for its classification [27]. In a similar vein, Huang et al. evaluated a DL approach for classifying AML, ALL, CML, and normal bone marrow using microscopy images and CNNs with transfer learning. They analyzed 104 bone marrow smear cases (18 normal, 53 AML, 23 ALL, 18 CML), first enhancing images with a perfect reflection algorithm and adaptive filtering. Three CNN architectures (Inception-V3, ResNet50, and DenseNet121) were trained on raw and preprocessed datasets. DenseNet121 on the preprocessed images showed the best performance, with accuracy improving from 74.8% to 95.3% after transfer learning, and class-wise accuracies of 90% (normal), 99% (AML), 97% (ALL), and 95% (CML). The authors conclude that this method offers faster, more objective, and more accurate leukemia diagnosis than manual microscopy [28].

Dese et al. developed an ML system to classify leukemia subtypes from peripheral blood smear images. Using 250 clinical slides from Jimma Medical Center plus an online ALL database, they applied preprocessing, K-means-based segmentation, feature extraction, and multiclass SVMs. The model automatically distinguished four subtypes - ALL, CLL, AML, and CML - and estimated WBC counts. For test datasets, accuracy, sensitivity, and specificity were 97.69%, 97.86%, and 100%, and for validation datasets, 97.5%, 98.55%, and 100%. WBC counting achieved 94.75% accuracy. Processing each case required less than one minute, compared with about 30 minutes for manual review, and increased diagnostic accuracy from roughly 70% to over 97%. The tool is designed for deployment in resource-limited sub-Saharan African settings, supporting early detection and standardized clinical decision-making [29].

Shanbehzadeh et al. compared eight ML algorithms to predict five-year survival in patients with CML using data from 837 cases. Clinical and laboratory variables were retrospectively collected, preprocessed, and split 70:30 into training and test sets, with minimal redundancy maximal relevance used to select key predictors. Spleen palpable, age, and unexplained hemorrhage emerged as the most influential features. Among eXtreme gradient boosting, multilayer perceptron, k-nearest neighbors, probabilistic neural networks, J-48, and SVMs, the SVM with radial basis function kernel performed best, achieving 85.7% accuracy, 86% sensitivity, 85% specificity, F-measure of 87%, and area under the curve (AUC) of 0.85 on selected features. The authors conclude that these models can support individualized prognostication and treatment planning in CML and guide more personalized follow-up [30].

Cheng et al. used multiple ML algorithms to identify prognostic biomarkers in acute myeloid leukemia using GEO and TCGA transcriptomic datasets. Differentially expressed genes were filtered, and survival analysis yielded 26 genes associated with overall survival. LASSO, RF, SVM-RFE, and XGBoost were applied, and the intersection highlighted three key genes: DNM1, MEIS1, and SUSD3. Pan-cancer analysis showed MEIS1 and DNM1 were highly expressed in AML, while MEIS1 and SUSD3 acted as risk factors and DNM1 as a protective factor for prognosis. These genes correlated with AML immune subtypes and several immune checkpoints. Validation in external cohorts and receiver operating characteristic (ROC) analysis confirmed their diagnostic and prognostic value, supporting ML-guided risk stratification and targeted therapy development. The study demonstrates how bioinformatics and ML refine precision oncology [31].

Nielsen et al. used various ML models, including logistic regression, RF, AdaBoost, and artificial neural networks (ANNs), to predict the risk of asparaginase-associated pancreatitis (AAP) in children with ALL. The models were trained on germline single-nucleotide polymorphisms (SNPs) and clinical data from 1,390 patients of European ancestry (205 with AAP) and validated on 274 patients. Models that combined 30 AAP-associated SNPs with age and sex achieved a strong predictive accuracy (ROC-AUC 0.80), which improved to 0.83 with an ensemble approach, though performance was lower in non-European patients (AUC 0.72). The predictive power further increased to an AUC of 0.86 by adding information on asparaginase dosing intensity and number of doses. This research supports using these models for personalized toxicity-risk assessment, informing prevention trials, and guiding safer decisions regarding re-exposure to asparaginase in survivors [32].

**Figure 2:**
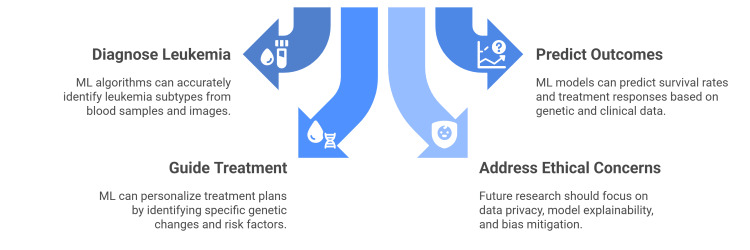
Use of AI in leukemia. Image credit: Created by authors based on [18-23].

The future of ML in leukemia diagnosis requires a shift from algorithm development to practical clinical implementation. Future research must prioritize the creation of large, high-quality, and heterogeneous datasets to address current reproducibility issues and small sample sizes. There is a critical need for prospective studies to validate these models in real-world settings. Additionally, efforts should focus on ensuring model explainability, addressing ethical concerns related to data privacy, and mitigating potential biases to enable safe integration into standard hematology workflows (Figure 2) [23].

Lymphoma

Lymphoma encompasses a group of biologically and clinically diverse diseases, usually presenting as non-painful swollen lymph nodes, often accompanied by systemic symptoms like fever, night sweats, and unexplained weight loss. Pathologically, lymphoma is primarily categorized into Hodgkin's lymphoma (HL) and non-Hodgkin's lymphoma (NHL). NHL includes several subtypes, such as mantle cell lymphoma (MCL), follicular lymphoma (FL), marginal zone lymphoma, and the most prevalent form, diffuse large B-cell lymphoma (DLBCL). Despite significant advancements in diagnostic techniques and the adoption of standardized treatments, which have markedly improved survival rates for lymphoma patients, NHL continues to impose a greater disease burden compared to HL [33].

In the last ten years, ML tools have enhanced the precision of diagnosing, analyzing genomics, proteomics, and histopathology in lymphoma, which has facilitated personalized treatment and increased survival rates [34].

Steinbuss et al. developed a deep-learning model (EfficientNet-based) for classifying histopathological whole-slide image patches from lymph nodes into three categories: tumor-free, nodal small lymphocytic lymphoma (SLL) or CLL, and nodal DLBCL. The model was trained on 84,139 patches and tested on an independent set of 16,960 patches, achieving an accuracy of 95.56%. The study concludes that automated, highly accurate classification of NHL subtypes using DL on histopathological images is feasible, with a call for future efforts focused on clinical integration [35].

Tagami et al. developed ML models utilizing texture analysis to distinguish between orbital and conjunctival mucosa-associated lymphoid tissue (MALT) lymphomas using hematoxylin and eosin-stained (H&E) histopathological images. The study analyzed tissue samples from 129 patients, extracting features from image patches at three different magnifications (×4, ×20, and ×40) to train seven distinct algorithms. Results indicated that the SVM with a linear kernel was the superior algorithm, achieving an average accuracy rate of 85%, with ×20 magnification providing the highest diagnostic precision [36].

Other authors investigated the potential of DL models to diagnose cervical lymphadenopathy using contrast-enhanced CT images, aiming to reduce reliance on invasive histopathological examinations. The study retrospectively analyzed 400 patients divided equally into four groups: granulomatous diseases, lymphoma, squamous cell tumors, and reactive hyperplasia. The researchers employed three DL architectures - ResNet50, NASNetMobile, and DenseNet121 - to classify the images. Among these, ResNet50 demonstrated the highest classification accuracy at 92.5%, followed by DenseNet121 at 90.62%. The authors concluded that while DL shows promise as a non-invasive diagnostic tool, further research with larger datasets is necessary [37].

Zhuang et al. developed an ML model to classify DLBCL patients based on a 7-mRNA signature. By analyzing genomic data from 1,143 samples, the researchers identified two distinct molecular subtypes: immune-enriched (IME) and cell-cycle-enriched (CCE). Among the seven algorithms tested, the SVM demonstrated the highest performance, using seven key genes (including SERPING1 and TIMP2) to achieve an accuracy of 88.6% and an AUC of 0.973. Validated across three external datasets, this model served as an independent prognostic factor, offering a reliable tool for guiding individualized clinical treatment [38].

Prognosis of Lymphoma

Creating a prognostic model for lymphoma can assist in anticipating how the disease will advance, evaluating associated risks, and determining the effectiveness of treatment, thereby enabling physicians to make better-informed treatment choices. Several factors can predict lymphoma outcomes, one of which is the total metabolic tumor volume (TMTV). TMTV quantifies the metabolically active volume of a tumor using whole-body fluorodeoxyglucose-positron emission tomography/computed tomography (FDG-PET/CT) and is recognized as a powerful, independent prognostic indicator for both HL and NHL [39]. 

Patient prognosis in DLBCL was assessed based on the extent to which the cancerous lesions had spread throughout the body. This spread was accurately determined by analyzing the findings from the initial PET/CT scans performed at the time of diagnosis [40]. Despite its importance for risk stratification in previously untreated MCL patients, TMTV is not currently considered the strongest independent predictor of outcome. Established prognostic factors such as the International Prognostic Index for MCL (MIPI) and the proliferation marker KI-67 still hold greater distinction in predicting patient prognosis and guiding therapeutic decisions. However, TMTV remains a valuable measure, providing complementary spatial information about tumor burden that can refine risk assessment when integrated with these other established clinical and biological markers [41].

Capobianco et al. showed that DL technique could automatically calculate TMTV in many DLBCL patients, with results matching expert measurements. The TMTV estimation proved to be a significant predictor of both progression-free survival and overall survival in these patients. Other researchers developed a novel prognostic model by incorporating clinical data. This new model demonstrated significantly superior validation compared to existing prognostic indicators for DLBCL, like the International Prognostic Index [42,43].

Treatment of Lymphoma

The growing use of ML algorithms is enhancing patient care by improving treatment selection and expanding options for diseases like lymphoma, where researchers are focused on developing targeted therapies. Key clinical challenges for lymphoma patients involve determining the optimal treatment plan and reducing the toxicity of chemotherapy and radiotherapy. The rapid development of new targeted drugs is enabled by recent advancements in genomics and AI. This shift toward a more individualized approach, known as precision or personalized medicine, has shown significant benefits for patients [44,45]. Rosenberg et al. utilized Next-Generation Sequencing (NGS) and electronic technologies to create a research framework. This framework introduces a new approach for assessing drug effectiveness, safety, pharmacokinetics, and pharmacodynamics. Consequently, this could speed up drug development and help achieve the objective of improving patient outcomes [46,47]. PET/CT is highly sensitive in detecting both primary and metastatic lymphoma, allowing for assessment of lesions, treatment effectiveness, and guidance for patient follow-up care. An AI method, the probability-corrected ensemble, has been shown to predict mortality in patients with DLBCL, aiding in evaluating treatment outcomes and optimizing chemotherapy. AI's impact on lymphoma extends to radiology, where it is used for diagnosis, anatomical measurement, and, crucially, for more effective risk stratification in HL, thus influencing treatment decisions. Furthermore, the integration of NGS and AI in lymphoma helps predict a patient's response to chemotherapy [5].

Multiple myeloma

Multiple myeloma (MM) is a cancer of plasma cells primarily affecting older individuals. Diagnosing and staging MM relies on a range of data, including serological, genetic, morphological, immunophenotypic, histological, and radiological information. ML has been investigated for its potential to identify early MM markers, which could guide treatment decisions and consequently influence recurrence rates and patient survival. Imaging is vital for detecting bone lesions, particularly lytic ones, which are critical for diagnosis, prognosis, and therapeutic planning, and ML offers utility in this specific area [48]. 

For instance, Xiong et al. investigated the utility of ML-based radiomics to differentiate MM from metastatic tumors in lumbar vertebra lesions. Retrospectively analyzing 107 patients, the researchers extracted 282 texture features from T1- and T2-weighted MRI images to train five distinct algorithms. The ANN model using T2-weighted images emerged as the most effective classifier, achieving an accuracy of 81.5% in distinguishing MM from metastases. However, the model showed only moderate performance in identifying specific metastasis subtypes (accuracy 64.8%), suggesting that while promising for general differentiation, further refinement is needed for subtype classification [49]. 

Guerrero et al. developed an ML model to predict undetectable measurable residual disease (MRD) in newly diagnosed MM patients by integrating tumor and immune biomarkers. Analyzing data from 487 patients across three clinical trials (GEM2012MENOS65, GEM-CESAR, and GEM-CLARIDEX), the researchers trained and validated a model that combined cytogenetics, tumor burden, and immune profiles. The algorithm successfully predicted MRD outcomes with approximately 71% to 72% accuracy across the cohorts. Notably, patients identified by the model as having a high probability of undetectable MRD exhibited superior five-year progression-free (80%) and overall survival (93%) rates [50].

## Conclusions

AI is rapidly transforming the diagnostic and clinical landscape of hematologic malignancies by enhancing accuracy, accelerating workflows, and supporting personalized treatment strategies. Across leukemia, lymphoma, and MM, ML and DL models have demonstrated strong performance in tasks such as subtype classification, prognostic modeling, automated image interpretation, and prediction of treatment response or toxicity. These technologies offer the potential to integrate complex clinical, genomic, radiologic, and histopathologic data into unified decision-support tools, ultimately improving risk stratification and enabling more precise, individualized patient care. Early successes, such as automated leukemia detection, DL-based lymphoma classification, radiomics-driven myeloma assessment, and AI-assisted toxicity prediction, highlight the significant clinical promise of AI in hematology.

However, meaningful translation of these advances into real-world practice requires addressing several ongoing challenges. Most current models rely on small, heterogeneous datasets and lack prospective, multi-center validation, limiting generalizability. Ensuring transparency, explainability, and fairness in AI systems is crucial to building clinician trust and avoiding biased outcomes. Robust data governance frameworks must also be established to safeguard patient privacy and support ethical integration into clinical workflows. Future efforts should prioritize high-quality dataset creation, standardized evaluation methods, and closer collaboration between clinicians, data scientists, and regulatory bodies. With these steps, AI can evolve from experimental tools into reliable, clinically embedded systems that genuinely enhance the management of hematologic malignancies.
